# *De Novo* Whole-Genome Sequence of *Pantoea latae* Strain AS1, Isolated from *Zamia floridana* Rhizosphere in Central Florida, USA

**DOI:** 10.1128/genomeA.00640-17

**Published:** 2017-07-13

**Authors:** Pushpa Lata, Subramaniam S. Govindarajan, Feng Qi, Jian-Liang Li, Santosh K. Maurya, Malaya K. Sahoo

**Affiliations:** a Department of Microbiology and Molecular Biology, University of Central Florida, Orlando, Florida, USA; b Analytical Genomics Core, Sanford Burnham Prebys Medical Discovery Institute at Lake Nona, Orlando, Florida, USA; c Applied Bioinformatics Core, Sanford Burnham Prebys Medical Discovery Institute at Lake Nona, Orlando, Florida, USA; d Sanford Burnham Prebys Medical Discovery Institute at Lake Nona, Orlando, Florida, USA; e Department of Pathology, Stanford University School of Medicine, Palo Alto, California, USA

## Abstract

*Pantoea latae* strain AS1 was isolated from the rhizophere of a cycad, *Zamia floridana*, in central Florida, USA. Here, we report the *de novo* whole-genome sequence of this strain, which consists of a total of 83 contigs spanning 4,960,415 bp, with a G+C content of 59.6%, and comprising 4,527 predicted coding sequences.

## GENOME ANNOUNCEMENT

The genus* Pantoea* is a group of rod-shaped Gram-negative bacteria that belong to the family *Enterobacteriaceae* (1). *Pantoea* has been isolated from a multitude of environments and harnessed for various purposes, *viz*., bioremediation and degradation of toxic herbicides, antibiotic production, and as a biocontrol agent against some plant pathogens (2, 3). In this study, *Pantoea latae* strain AS1 was isolated from the rhizosphere of a cycad, *Zamia floridana*, in central Florida, USA. Here, we report the *de novo* whole-genome sequence of *Pantoea latae* strain AS1. The genomic DNA was isolated using the QIAamp DNA minikit (Qiagen, Germantown, MD), according to the manufacturer’s instructions. DNA quality was checked by a NanoDrop spectrophotometer (Thermo Scientific) and quantitated by a Qubit 2.0 fluorometer (Life Technologies, Inc.) and Agilent 2100 Bioanalyzer (Agilent Technologies, Santa Clara, CA). The genomic DNA was fragmented and tagged with sequencing adapters in a single-tube enzymatic reaction using the Nextera XT DNA library preparation kit (Illumina, San Diego, CA). The resulting library was sequenced using the Illumina MiSeq sequencing platform (Illumina) and generated 3.6 million reads for *Pantoea latae* strain AS1. The reads were assembled using the Minia version 2.0.3, with default parameters and a k-mer size of 121 (4). A total of 83 contigs were obtained, and the *N*_50_ of the contigs was 204,505 bp. The maximum length of a contig was 376,349 bp, with a total genome length of 4,960,415 bp. The G+C content of the genome was 59.6%. The protein-coding regions were predicted by the NCBI Prokaryotic Genome Annotation Pipeline (release 2013; https://www.ncbi.nlm.nih.gov/genome/annotation_prok/). In total, 4,527 coding genes were identified in the 4.9-Mb genome, showing 75% symmetrical identity with the *Pantoea septica* genome. Seventy-eight tRNA genes, 29 rRNAs (14 5S rRNAs, 2 16S rRNAs, and 13 23S rRNAs), 9 noncoding RNA (ncRNA) genes, the copper resistance protein CopD, nitrate assimilation pathway proteins, and antibiotic synthesis proteins have putative functions assigned on the basis of the annotation.

### Accession number(s).

This whole-genome shotgun project has been deposited at DDBJ/ENA/GenBank under the accession no. MWUE00000000. The version described in this paper is MWUE01000000.
